# Metabolomic and proteomic applications to exercise biomedicine

**DOI:** 10.1515/teb-2024-2006

**Published:** 2024-03-21

**Authors:** Daniel J. Wilkinson, Hannah Crossland, Philip J. Atherton

**Affiliations:** Centre of Metabolism, Ageing & Physiology (CoMAP), Medical Research Council/Versus Arthritis UK Centre of Excellence for Musculoskeletal Ageing Research (CMAR), School of Medicine, University of Nottingham, Royal Derby Hospital, Derby, UK

**Keywords:** OMICs, stable isotope tracers, exercise, muscle

## Abstract

**Objectives:**

‘OMICs encapsulates study of scaled data acquisition, at the levels of DNA, RNA, protein, and metabolite species. The broad objectives of OMICs in biomedical exercise research are multifarious, but commonly relate to biomarker development and understanding features of exercise adaptation in health, ageing and metabolic diseases.

**Methods:**

This field is one of exponential technical (i.e., depth of feature coverage) and scientific (i.e., in health, metabolic conditions and ageing, multi-OMICs) progress adopting *targeted* and *untargeted* approaches.

**Results:**

Key findings in exercise biomedicine have led to the identification of OMIC features linking to heritability or adaptive responses to exercise e.g., the forging of GWAS/proteome/metabolome links to cardiovascular fitness and metabolic health adaptations. The recent addition of stable isotope tracing to proteomics (‘dynamic proteomics’) and metabolomics (‘fluxomics’) represents the next phase of state-of-the-art in ‘OMICS.

**Conclusions:**

These methods overcome limitations associated with point-in-time ‘OMICs and can be achieved using substrate-specific tracers or deuterium oxide (D_2_O), depending on the question; these methods could help identify how individual protein turnover and metabolite flux may explain exercise responses. We contend application of these methods will shed new light in translational exercise biomedicine.

## Introduction

Translational exercise biomedicine (TEB) can be thought of as a field bridging the interface of discovery sciences and applied sciences. Use of ‘OMICS in exercise biomedicine has many purposes, such as linking OMIC datasets to underlying physiology, developing novel biomarkers, and informing on sports performance and health. In particular, there is a core interest in discovering ‘molecular transducers’ of exercise adaptation and established health benefits (Sanford et al., 2020 Cell 181, 1464); as there is in back-linking OMIC data to genotypes e.g., quantitative trait loci (QTL), across biological order i.e., DNA modification, RNA species, peptidomes, proteomes and metabolomes.

The molecular biology revolution in pre-clinical models paved the way for tools permitting molecular modification of gene expression, permitting mechanistic linkage of genotypes/phenotypes, i.e., developmental, and inducible models of over and under-expression. In less tractable experimental medicine studies in humans, where complex outbred genomes exist, the most potent tools to uncover links across biological orders to complex phenotypes – is ‘OMICs [1]. In terms of exercise biomedicine, each of these levels has been studied, with arguably the greatest focus of the past 25+ years being on mRNA (transcriptomics) due to the excellent coverage (now >44,000 protein-coding genes, alongside >40,900 non-protein coding transcripts e.g., via Affymetrix) offered by microarray approaches, and more recent repurposing of next generation sequencing (NGS) to RNASeq analysis. While beyond the scope of this review, it is worth noting the relative merits of these approaches [2], also the advent of spatial transcriptomics [3], which offers tissue level resolution of transcript distribution (and proteins) in relation to co-localization to structures of scientific interest. More recently in exercise biomedicine, there has also been a focus on how exercise may alter gene expression through post-translational modification of DNA such as methylation and acetylation (silencing/activating) gene expression both acutely [4, 5] affecting, and perhaps leaving sustained molecular footprints, termed muscle memory [6]. Readers are referred to several review articles [2, 7], [8], [9], [10] if interested in methodologies to confer study at these biological levels.

Metabolomics and proteomics, the focus of this review, are developing fields, and may be targeted (or semi-) or untargeted in nature. For instance, targeted meaning chosen “panels” of metabolites or proteins using technologies, such as nuclear magnetic resonance (NMR) or mass spectrometry (MS) (discussed in more detail later) or commercial platforms such as O-link and Somalogic, for proteomics (detecting ∼5,000 and ∼11,000 proteins, respectively). While MS remains the gold standard, there are relative merits of each technique [11]. Increasingly high-resolution MS instrumentation coupled to optimization of sample pre-processing, is such that metabolome and proteome coverage continue to improve. Most recently, the opportunity of combining stable isotope tracers (SIT) with proteomics and metabolomics using MS approaches (we hereon in term this “SITOMICS”) offers the potential to, e.g., map dynamics of individual protein synthesis (rather than point-in-time concentrations) with metabolite flux through, for instance, ATP-producing pathways. While in its relative infancy *in vivo* at least, ‘SITOMICs’ will offer the exciting opportunity to study the dynamics of exercise biomedicine as it pertains to e.g., exercise adaptation, non-responders, health benefits and QTL/multi-OMIC approaches.

Physical activity leads to widespread beneficial changes in metabolic, cardiovascular, and immune pathways among others, but the mechanisms by which exercise benefits human health remain incompletely defined. The advancement of ‘OMICS technologies continue to enable development of our existing knowledge surrounding the molecular responses to exercise in an attempt to increase our mechanistic understanding of the adaptive changes to exercise training. Moreover, wide-scale metabolite and/or protein profiling has the advantage of gaining a more comprehensive and unbiased overview of energy metabolism with exercise, as opposed to classic biochemical techniques that target single pathways. The following sections of this review will outline the current state of ‘OMICS (specifically on metabolomic and proteomic studies) in the field of exercise biomedicine, particularly focusing on human studies and health-related outcomes.

## MS-based metabolomics and proteomics

**Figure 1:**
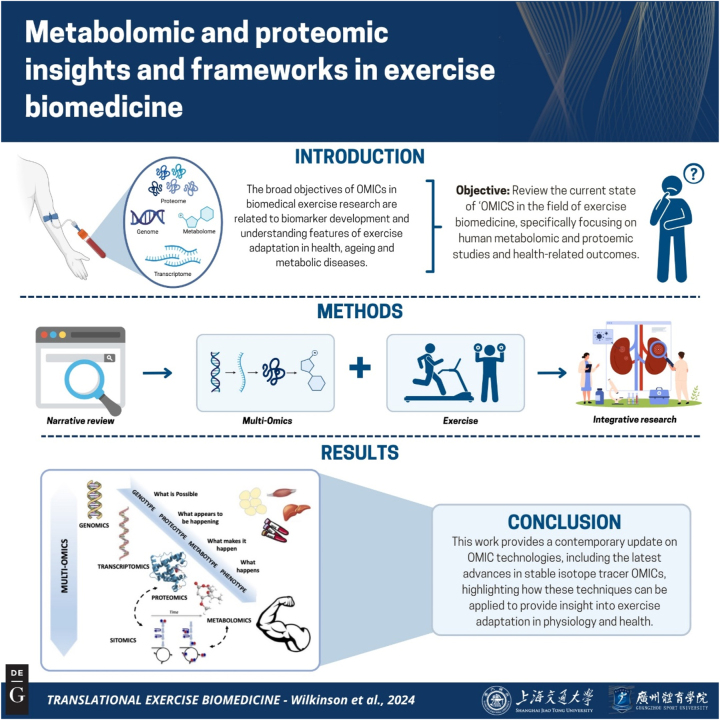
Graphical representation of the study. Keys points: 1) This work reflects the increasing use of post genomic/transcriptomic methodologies in translational exercise biomedicine, which as such, focuses on proteomics and metabolomics. This work aims to be translational via providing basic science information to inform clinical and/or applied exercise biomedicine – vis-à-vis illuminating current, and future steps of OMIC’s i.e., combining stable isotopes with OMIC’s to track the dynamics of metabolism; 2) This is a narrative review encapsulating key literature in what is a niche (technologically at least) yet exponentially growing field in exercise biomedicine. This work explores the result of application of OMIC techniques in exercise biomedicine as they stand and what the future may look like in terms of biomarker development and mechanistic insights in this field; 3) This work provides a contemporary update on OMIC technologies and includes core relevant data to illustrate how these techniques have been applied and provided insight into exercise adaptation in physiology and health. Figure created with BioRender.

As the final two endpoints to the ‘OMICs continuum (see Figure 1), measurement of the proteome and metabolome of key biological systems/tissues, should in theory provide the most accurate insight into the biological mechanisms driving observed phenotypes [12]. As such, proteomics and metabolomics provide the most translatable potential of any ‘OMICs measures, particularly from a medical or clinical standpoint [12]. The increased availability, access and use of these proteomics and metabolomics technologies has largely been possible due to the rapid hardware and software developments, particularly in the field of MS [13]. It is now possible utilising high-resolution MS (HRMS) to measure the abundance/relative concentrations of >14,000 proteins and >10,000 metabolites in a single biological sample in as little as 0.5 h [14]. This provides a wealth of data that could provide insights into aspects regulating metabolism that were previously unknown and may produce targets for future manipulation. This is precisely why these approaches have become popular within the fields of exercise biomedicine.

Metabolomics experiments can be either classed as untargeted or targeted, and the use of either depends entirely on the desired outcome or research questions being asked. The aim of untargeted metabolomics is to measure the overall abundance of as many metabolites as possible (up to 10,000) within a single sample at once using either a single analytical instrument or combination of analytical instruments. This technique generates high volumes of unbiased data, which are not generally led by *a priori* hypotheses, with the aim to look at patterns or changes in abundance of groups or networks of metabolites that may help to explain the biology underlying phenotypic change driven by the experiment [15]. Untargeted metabolomics, unfortunately due to its nature of trying to measure as many metabolites as possible is only semi-quantitative relying on changes in relative abundance/intensity of metabolites rather than absolute concentrations. As such, untargeted metabolomics is generally considered the exploratory or hypothesis generating phase of the metabolomics pipeline [15]. From this untargeted data key research questions about the data collected can be generated, and targeted metabolomics analyses can then be focused on trying to answer these new questions. Targeted metabolomics hence looks at measuring the absolute concentration of groups or panels of key metabolites of interest as a focus of the research questions generated from this prior untargeted work. Unlike untargeted metabolomics, targeted metabolomics provides data on a small number (10–100 s) of focused metabolites enabling a high level of precision and accurate measures of absolute concentration [15].

The two types of analytical instrumentation used for metabolomics are either Nuclear Magnetic Resonance Spectroscopy (NMR) or Mass Spectrometry. NMR generates spectroscopic data from the behaviour of the atomic nuclei of elements which make up the metabolites within a sample placed in a varying magnetic field (for more information see Bothwell and Griffin [16]). It is a highly reproducible technique, which can provide precise and detailed information on metabolite concentrations and chemical structure making metabolite identification relatively simple [17]. It also requires minimal sample preparation and is a non-destructive technique, meaning the sample is not lost and can be reused. However, the extent of metabolite coverage is limited (a few hundred metabolites maximum for untargeted runs) with NMR, and its dynamic range in terms of low abundant metabolites is also restricted [18]. Mass spectrometry in comparison generates spectral data from the mass to charge ratios of ionised molecules detected under high vacuum [19]. While the sample requires more preparative steps and is not reusable once analysed, when combined with hybrid separation techniques such as Gas Chromatography (GC) and/or Liquid Chromatography (LC), MS can provide a far deeper coverage of the metabolome across a wider dynamic range of abundances [18]. As such MS is generally considered the gold standard in most current metabolomics applications.

## Metabolomics in exercise biomedicine

An early investigation into profiling the exercise metabolome was conducted by Pohjanen et al. [20]; who measured over 400 serum metabolites pre- and post-exercise in trained, young males (four tests of 90 min standardized ergometer-cycling, with one week between each test). They found that 34 metabolites were significantly different from pre- to post-exercise, with glycerol and asparagine being identified as potential exercise biomarkers. This study highlighted that untargeted GC-MS-based metabolomics can offer a detailed, untargeted approach to studying the metabolic changes in response to exercise interventions. To investigate changes at the tissue level, a study published by Zagatto et al. [21] profiled intramuscular metabolites using 1H NMR spectroscopy, in humans at rest and following high-intensity cycling exercise (healthy, physically active males underwent a graded exercise test and a supramaximal effort to exhaustion). The highest impacted metabolic pathways included pyruvate metabolism, TCA cycle, and mitochondrial electron transport chain, as well as fatty acid oxidation and amino acid metabolism. These findings are in-keeping with what would be predicted based on energetic stress.

There is in fact now a collective body of literature investigating metabolomic changes in response to a range of exercise types and intensities/duration (see Table 1 for a summary of the key studies discussed). In a study by Nieman et al. [22]; male and female long distance runners (aged 19–45 years) ran for 2 h/day for three days at ∼70 % VO2max. In collected blood samples, there were significant increases in metabolites related to lipid and amino acid/peptide metabolism immediately following the three-day exercise period, and following a 14 h rest period, it was observed that many of the metabolites measured were still not restored to pre-exercise levels. In further work [23], blood samples were collected at rest from elite athletes from different sports, divided into high endurance, moderate endurance, high power, and moderate power athletes. Untargeted metabolomics analyses by MS combined with ultra-high performance liquid chromatography MS (UPLC-MS/MS) revealed significant associations of circulating diacylglycerols and eicosanoids with high endurance, as well as several metabolites involved in sex steroid biosynthesis. High-power athletes had higher levels of phospholipids and xanthine metabolites than moderate power athletes. This highlights distinct metabolic profiles between athletes of different disciplines, i.e., related to steroid biosynthesis and fatty acid metabolism.

**Table 1:** Key details of metabolomics-based exercise studies described in the review.

Authors (year)	Study population	Intervention protocol	Biological sample	Primary findings
Pohjanen et al. [20]	Twenty four healthy young, trained males	Four tests of 90 min standardized ergometer-cycling (one week between each test)	Serum	Thirty four metabolites altered pre to post exercise (glycerol and asparagine highlighted as potential biomarkers)
Nieman et al. [22]	Fifteen long distance runners (seven males, eight females, 19–45 years)	Running for 2.5 h/day for three days at ∼70 % VO2max	Serum	Sustained changes in metabolites related to lipid and amino acid/peptide metabolism
Al-Khelaifi et al. [23]	One hundred ninety one elite athletes (171 males and 20 females)	Samples collected at rest (high/moderate endurance, and high/moderate power athletes)	Serum	Distinct metabolic profiles between high-power and high-endurance athletes related to steroid biosynthesis and fatty acid metabolism
Pellegrino et al. [24]	Fourty healthy young, physically active males and females	Acute bouts of either aerobic (45 min of aerobic cycling) or anaerobic (10-repetition max testing for seven lower-body exercises) exercise	Serum	Aerobic exercise showed greater use of fatty acids, while resistance exercise showed higher glycolytic flux and purine turnover
Wu et al. [25]	Eight young, healthy males	Three bouts of cycling at full force for 30 s	Serum	Changes in metabolites related to amino acid, lipid and glucose metabolism
Gaitán et al. [26]	Twenty five middle-aged adults at risk from Alzheimer’s disease	Twenty six weeks of aerobic exercise training	Serum	Increased PUFA and lysophospholipids, and decreased ceramides, sphingolipids and phospholipids
Morville et al. [27]	Ten young, healthy males	Crossover study with endurance exercise (1 h at 70 % VO2max) or 1 h strenuous high-volume resistance exercise (one week between each bout)	Plasma	Distinct metabolic pathways impacted by each exercise mode (e.g. BCAA metabolites altered by resistance exercise, several acylcarnitines altered by endurance exercise)
Nayor et al. [28]	>400 middle-aged males and females	Maximal effort cardiopulmonary exercise testing	Plasma	Altered aerobic and anaerobic respiration, glycolytic flux and mitochondrial fatty acid oxidation
Li et al. [29]	Nineen young active adults (13 males and six females)	Acute endurance exercise (90 min outdoor run at an effort of 75–80 % of their heart rate maximum)	Plasma and cerebrospinal fluid	Many metabolites in CSF impacted by 1 h endurance exercise, purines and pyrimidines most significantly altered
Zagatto et al. [21]	Seven healthy young, physically active males	High-intensity cycling (graded exercise test and a supramaximal effort to exhaustion)	Skeletal muscle tissue	Impacted pyruvate metabolism, TCA cycle, and mitochondrial electron transport chain, as well as fatty acid oxidation and amino acid metabolism
Mendham et al. [30]	Young black South African women, BMI of 30–40 kg/m^2^	Twelve weeks combined aerobic and resistance exercise, moderate-vigorous intensity for 40–60 min, four sessions per week	Skeletal muscle tissue	Training altered specific lipid intermediates, associated with increased mitochondrial content but not whole-body insulin sensitivity

Morville et al. performed untargeted metabolomic profiling of plasma samples from a cross-over study with young, healthy males involving endurance and resistance exercise (one-week rest between bouts, with samples taken pre, after 1 h exercise, and during a 3 h recovery phase) [27]. UPLC-MS/MS was performed on plasma samples, which resulted in the detection of >800 metabolites. They found 666 metabolites were altered with endurance exercise while 708 were altered with resistance exercise, a subset of which being specific to each mode of exercise. In general, a greater number of metabolites were upregulated with endurance than resistance exercise, although there was a greater magnitude of response with the upregulated metabolites with resistance exercise. Many breakdown metabolites of BCAA showed marked responses to resistance exercise, as well as TCA cycle and glycolytic metabolites, while lipid-related metabolites were substantially altered by endurance exercise, including several acylcarnitines and the ketone metabolite β-Hydroxybutyric acid (BHBA).

Serum metabolite responses to acute aerobic or anaerobic exercise were also assessed in a recent study [24]. Both forms of exercise in young, physically active males and females (which consisted of either 45 min of aerobic cycling or 10-repetition max testing for seven lower-body exercises) resulted in significant alterations in metabolite abundance immediately following exercise (with increases in purine metabolism, substrate catabolism and mobilization, and inflammatory mediators), which were returning towards baseline after 1 h post exercise. There were also differences between the modes of exercise; aerobic exercise showing greater use of fatty acids, and resistance exercise demonstrating higher glycolytic flux and purine turnover. Overall, the above studies demonstrate that research into exercise-responsive metabolites using metabolomics can increase understanding of underlying adaptations occurring in response to exercise, as well as identifying potentially clinically relevant biomarkers that could aid in health research.

Several studies have also used metabolomics to help understand how responses to acute exercise elicit adaptive benefits in multiple organs, such as the brain and heart. In a study by Li et al. [29]; metabolite analyses were performed on plasma and cerebrospinal fluid (CSF) from young active males and females following acute endurance exercise (which consisted of a 90 min outdoor run at an effort of 75–80 % of their heart rate maximum). Many metabolites in CSF were impacted by 1 h endurance exercise, with purines and pyrimidines most significantly altered, and pathways included those linked to dopamine, purines, amino acids and BCAA. In relation to this, there is evidence that exercise training could delay or prevent the progression of Alzheimer’s disease (AD). Since there is a lack of systemic biomarkers linking exercise effects on brain function and relevant metabolic responses, a study by Gaitán et al. [26] aimed to address this by measuring the effects of 26 weeks of aerobic exercise training in late middle-aged adults at risk for AD on metabolomic markers of brain health. Following 26 weeks of training, increases in PUFA and lysophospholipids, and decreases in ceramides, sphingolipids and phospholipids were observed. Ceramides and sphingolipids have previously been proposed as AD biomarkers and linked to cognitive function and mood regulation [31, 32]. Thus, systemic metabolite markers that can assess the effects of exercise on AD-related outcomes offer potential for insights in disease progression, and development of therapeutics.

Reduced mitochondrial capacity and lipid accumulation can lead to accumulation of lipid intermediates in skeletal muscle, while exercise training in individuals with type 2 diabetes can improve glycaemic control and insulin sensitivity, as well as mitochondrial function/content [33], [34], [35]. Mendham et al. [30] sought to evaluate how combined aerobic and resistance exercise training in young women with obesity might influence changes in specific intermediates of phospholipids, DAGs and ceramides, and how such changes may be linked to improvements in insulin sensitivity and mitochondrial function. Exercise training (12 weeks of combined aerobic and resistance exercise at a moderate-vigorous intensity for 40–60 min and four sessions per week) significantly altered the lipid profile of skeletal muscle, with increases total phosphatidylcholine and phosphatidylethanolamine. These changes were associated with increased mitochondrial content (as measured by citrate synthase protein content), but not with increased whole-body insulin sensitivity (measured through intravenous glucose tolerance tests). A recent study also assessed the effects of high-intensity exercise on the metabolome of the myocardium [25]. There were similar alterations in metabolites within the myocardium of rats following high intensity exercise, and in serum from young healthy male participants who performed three bouts of cycling at full force for 30s, with changes related to amino acid, lipid and glucose metabolism (lineolic acid metabolism, pyrimidine metabolism, fructose and mannose metabolism, nicotinate and nicotinamide metabolism). Studies such as these can increase our understanding of the impact of high intensity exercise on the myocardium and could be useful for use in the field of exercise biomedicine and disease prognosis.

In a larger scale study by Nayor et al. [28]; metabolites were profiled in blood samples taken before and after an acute bout of exercise (maximal effort cardiopulmonary exercise testing), on over 400 participants (middle-aged males and females). The aims of the study were primarily to investigate the metabolic responses to exercise, and how these responses may help to explain the physiological mechanisms underlying the cardiovascular benefits of exercise. As expected, a variety of biological pathways were represented in the altered metabolites following exercise, including increased aerobic and anaerobic respiration, altered glycolytic flux and mitochondrial fatty acid oxidation. Exercise-induced changes in metabolite concentrations were also subject to heterogeneity across individuals, for example, the metabolite dimethylguanidino valerate (DMGV) decreased following exercise, to a greater extent in lean individuals than participants with obesity/overweight. Analyses also revealed that abundance of some pre-exercise metabolites were associated with physiological measures of fitness (peak VO2, exercise blood pressure). These studies highlight how metabolomics can be beneficial in identifying target pathways that could potentially reduce toxic lipid accumulation in skeletal muscle in obesity and type 2 diabetes. In summary, metabolomics will continue to advance the field of exercise biomedicine.

## Proteomics in exercise biomedicine

Exercise training also confers adaptations to proteins involved in a wide range of biological functions, and proteomics analyses has begun to improve our understanding of the underlying molecular alterations that lead to skeletal muscle remodelling with exercise. MS-based proteomics methods have been used to study the skeletal muscle proteome, however, there have to-date been few studies published that have focused on global impacts of exercise training on the skeletal muscle proteome, particularly in relation to pathophysiological conditions (such as type 2 diabetes). There is an added complexity of proteomics analyses, since many human genes undergo alternative splicing, which can produce different proteins with distinct functions. Protein diversity is also increased with the existence of post-translational modifications (PTMs), such as phosphorylation, ubiquitination, and glycosylation, which exponentially increases the complexity of the proteome. Detailed proteomic analysis of skeletal muscle is also a challenge since around 50 % of the total protein consists of highly abundant contractile and metabolic proteins, making detection of low abundant proteins challenging (the same is true of blood plasma/serum with saturating albumin/IgG’s). Nevertheless, investigations into proteomic responses with exercise and how they link to functional and phenotypic changes have great potential for the field of exercise biomedicine.

In a study by Holloway et al. [36]; six weeks of high-intensity interval training in recreationally active males was used to study the global effects of exercise training on the proteome in human muscle. The training programme consisted of three sessions a week with 6 × 1 min bouts at VO2max combined with 4 min bouts at 50 % VO2max. Two-dimensional gel electrophoresis was used in combination with iTRAQ labelling and LC-MALDI MS/MS on muscle biopsies taken both pre and two days post training. They found that 20 proteins were differentially expressed following the six weeks training, including modulation of troponin T and muscle creatine kinase. More recently, work by Schild et al. [37] evaluated the effects of acute endurance exercise on global protein responses in vastus lateralis muscle from young, male untrained individuals and endurance-trained athletes. Analysis with LC-MS/MS revealed that 92 proteins were significantly different between trained and untrained groups, with oxidative phosphorylation and TCA cycle proteins being the most altered, intuitively. The acute bout of exercise resulted in significant depletion of enzymes related to energy metabolism, particularly in the trained athletes. These results confirm that metabolic and functional adaptations are key features of acute and long-term exercise training in human skeletal muscle.

A further study by Hody et al. [38] investigated the proteomic response in rectus femoris muscle of trained and untrained individuals (young males) after two acute eccentric exercises (three sets of 30 maximal contractions of the quadriceps muscle). Exercise tests were performed six weeks apart, with the trained group performing five sessions of progressive peak torque training (50–90 %) prior to the second eccentric exercise test. In both groups, protein abundance of MHC isoforms and other contractile proteins were reduced following the first and second exercise test, as measured by 2D-DIGE analysis. Increased proteome changes were also observed following the second eccentric exercise test, which was also associated with reduced markers of muscle injury (muscle soreness, plasma creatine kinase), indicating that eccentric training can modulate the proteome in relation to protection against muscle injury and soreness.

Older age (and associated non-communicable diseases) is associated with an impaired capacity for muscle adaptation in response to exercise training [39], and to date, there is a lack of knowledge regarding proteomic adaptations to exercise training in relation to ageing and why these impairments may be occurring. Robinson et al. [40] performed proteomic analyses in muscle from young (18–30 years) and older (65–80 years) adults (both male and female) before and after 12 weeks of exercise training (72 h after the last bout of exercise). Proteome analysis at baseline revealed reduced protein abundance in older adults, particularly in mitochondrial proteins. Both RET and HIIT upregulated pathways that indicated improved translational capacity, such as aminoacyl-tRNA synthesis and tRNA aminoacylation. More recently, work from our group has also sought to evaluate proteomic features of muscle adaptation to RET in younger vs. older individuals [41]. Muscle biopsies were collected from young and older adults (recreationally active males and females) pre and post 20 weeks RET (consisting of three sessions per week, where the first four weeks had a training intensity increase from 40 to 60 % 1-repetition maximum (1-RM), with the remaining 16 weeks set at 70 % 1-RM). For each condition, muscle sarcoplasmic fractions were pooled, to obtain average proteomic responses across conditions, which were analysed by iTRAQ labelling combined with tandem mass spectrometry. Responses to RET were different for young vs. older adults, with proteins predominantly related to cytoskeleton and focal adhesion being upregulated in young adults, while in older adults RET induced an upregulation of proteins related to glucose metabolism and mitochondrial function, supporting previous findings. In another study, the effects of exercise on the proteome in vastus lateralis muscle was investigated in individuals with type 2 diabetes [42]. Participants were trained for four weeks, with five days of cycling per week (three days at 55 % of maximal workload and two days of 6 × 5 min at 70 % of maximal workload). While training had no effect on body weight or body mass index, waist circumference was significantly reduced in patients with type 2 diabetes and maximal workload was increased by 15 %. Proteomic analysis (HPLC-ESI-MS/MS) of the effects of exercise training revealed the majority of altered proteins being related to mitochondrial function, with TCA cycle, electron transport chain, and beta-oxidation-related proteins. These studies (see Table 2 for a summary of the key studies discussed), whilst limited, contribute to our understanding of the molecular changes underlying impaired muscle adaptation with RET due to ageing or disease at the proteomic level, and provide focus for future researchers to investigate these observed differences in greater detail.

**Table 2:** Key details of proteomics-based exercise studies described in the review.

Authors (year)	Study population	Intervention protocol	Biological sample	Primary findings
Holloway et al. [36]	Five young, physically active males	Six weeks HIIT (three sessions a week with 6 × 1 min bouts at VO2max combined with 4 min bouts at 50 % VO2max)	Skeletal muscle tissue	Twenty proteins differentially expressed following training, including troponin T and muscle creatine kinase
Schild et al. [37]	Five young, untrained males and five endurance-trained athletes	Acute endurance exercise on bicycle ergometer for 60 min 80 % VO2max	Skeletal muscle tissue	Ninety two proteins different between trained and untrained groups, including oxidative phosphorylation and TCA cycle proteins
Hody et al. [38]	Young, sedentary (5) or moderately active (5) males	Two acute eccentric exercises tests six weeks apart (trained group performed five eccentric training sessions between tests)	Skeletal muscle tissue	MHC isoforms and other contractile proteins were reduced in both groups
Robinson et al. [40]	Young (18–30 years) and older (65–80 years) males and females	Twelve weeks HIIT (combined cycling and treadmill walking) or resistance training (lower and upper body exercises (four sets of 8–12 repetitions) two days per week)	Skeletal muscle tissue	Both RET and HIIT upregulated aminoacyl-tRNA synthesis and tRNA aminoacylation
Deane et al. [41]	Eight young (four male, four female), eight older (six male, two female) recreationally active adults	Twenty weeks RET (three sessions per week, first four weeks from 40 to 60 % 1-RM, remaining 16 weeks set at 70 % 1-RM)	Skeletal muscle tissue	Proteins related to cytoskeleton and focal adhesion altered in young adults, proteins related to glucose metabolism and mitochondrial function in older adults
Hussey et al. [42]	Six middle-aged patients with type 2 diabetes	Four weeks training, five days of cycling per week (three days at 55 % of maximal workload and two days of 6 × 5 min at 70 % of maximal workload	Skeletal muscle tissue	Training altered proteins related to mitochondrial function, TCA cycle, electron transport chain, and beta-oxidation-related proteins

## Multi-OMICs – greater insight through multiple measures

Proteomics and metabolomics, based on their positions in the OMICs continuum (see Figure 1) should provide the greatest of level of insight of any one of the OMICs technologies singly. With access to these measures becoming easier and costs continually decreasing, the concept of multi-OMICs (the measure of multiple levels of the OMICs continuum within a single sample) has started to become a reality. This provides insight into what is changing from a gene/transcript level down to a single metabolite all at once within the same sample, giving a wealth of data to infer towards the biological mechanisms driving phenotypic responses to exercise across multiple organs. Although still in its infancy in exercise biomedicine Contrepois et al. used a longitudinal multi-OMICS approach (targeted and untargeted metabolomics, lipidomics, proteomics and transcriptomics) to profile plasma and PBMCs before and after acute cardiopulmonary exercise, with the aim of fully characterizing the system-wide molecular responses to an acute bout of exercise [43]. Analyses identified clusters of molecules that exhibited certain patterns of change (e.g. rapid increases following a return to baseline, or delayed changes with a return to baseline during the recovery period). Molecules linked to anaerobic metabolism, fatty acid oxidation and immune responses exhibited rapid increases and return to baseline, while many amino acids, including the BCAA, decreased with exercise without returning to baseline within the 1 h recovery phase. In this study, participants had variable levels of insulin resistance. The authors observed that certain FFA increased post-exercise early on in insulin sensitive participants, but levels remained low in insulin resistant participants, while carbohydrate and amino acid metabolic responses were greater in the insulin sensitive cohort. This work highlights the benefits (and future application) of using multi-OMICS approaches for the identification of molecular pathways associated with responses to exercise. It should also be noted that for the continued effort to develop biomarkers for use in exercise and health research, more validation studies likely need to be performed in this area. A key area for future research should include replication of changes in metabolites/proteins in independent studies, thus ensuring such observations are robust and not simply reflective of experimental/biological variation.

## Stable isotope tracer ‘OMICs (“SITOMICS”) in exercise biomedicine

While being highly informative, traditional proteomics and metabolomics represents static steady state concentration/abundance, typically, at a single timepoint thus providing only a snapshot of the system [44]. This remains the case when measuring repeated temporal samples: metabolism is a highly dynamic process, with rates of metabolic flux/activity and turnover of proteins often undergoing rapid change in response to physiological perturbation despite minimal changes to overall concentration or abundance, with concentrations and activity not always aligning [45]. Therefore, traditional measures of the metabolome/proteome inevitably miss important information, key to inferring mechanistic understanding, and which helps to explain the relatively slow translation of OMICs outcomes to clinical implementation [46]. However, this obstacle can potentially be overcome. Through the introduction of stable isotope tracers (SIT) alongside traditional measures of proteomics and metabolomics, termed “dynamic proteomics” and “fluxomics” respectively, another level of dimensionality can be determined enabling the measurement of flux/activity/turnover, alongside concentration and abundance [44], to permit greater translational potential. While the use of stable isotopes to measure metabolic flux, activity or turnover has been performed experimentally for close to a century now [19], the ability to monitor stable isotope incorporation within multiple metabolite or protein networks within a single experiment or analyses has become possible primarily due to recent hardware development. The resolving power of MS instrumentation (which is generally defined as the mass of the analyte divided by the smallest mass difference that can be detected by the instrument) in particular has improved where it is now possible for most benchtop high resolution MS to have resolving power of anything from 20,000 – 480,000, with larger FT-ICR-MS instruments capable of greater than 1,000,000 resolution [47]. This level of resolution not only allows separation and identification of compounds of very similar accurate molecular mass, but also precise measures of isotopomer labelling in these compounds, in addition to the separation of isotopologues of different elemental species in the same compound (i.e., the label provided from 15N, 13C and deuterium can all be detected at once [48, 49]). This means that with analyses of a single metabolomics or proteomics sample, data on abundance/concentration in addition to isotopic labelling of several thousand compounds is possible. As a result, fluxomics and dynamic proteomic applications are subject to fast moving development.

## Metabolomic fluxomics and exercise biomedicine – the future?

The metabolism of a living cell or organism is a highly complex process, and the benefits of inclusion of SIT within a metabolomics experiment should be abundantly clear. While a certain treatment, exposure to disease or physical exercise may perturb metabolite levels to different degrees, many metabolites are involved in a multitude of different metabolic networks; deciphering through which network these changes in concentration have been driven is difficult in metabolomics [49, 50]. Taking something simple like glucose, its relationship with other molecules is so complex, that changes in the metabolic rates of any one or all of these may be responsible for a small change in concentration. Yet if a stable isotope tracer label is introduced, we may trace where this label is going or coming from on the glucose and infer which metabolic networks could be driving this perturbation. A simple example of this is measuring levels of labelling on carbon positions 2 and 5 on the glucose molecule; in doing so, we can determine whether the increase in glucose may be coming from gluconeogenesis or glycogenolysis, enabling us to infer causal interpretation of metabolic change [51, 52]. In addition, there is the potential for SIT to provide information of which compartments or pools within a cell or organism may be contributing to these metabolomic changes. Fan and colleagues provide a description of this, whereby the positional labelling of lactate and citrate can determine which pools of pyruvate within the cell might be being utilised at any one time [50].

Whilst SIT resolved metabolomics can provide more detailed metabolic insights, there have been few attempts to implement these techniques within the field of exercise biomedicine. There have been many uses of SIT in a targeted fashion to elicit information on aspects of glucose, lipid, AA and energy metabolism (for more information see the following excellent reviews by refs. [19, 53, 54]) yet true stable isotope resolved metabolomics remains limited to mainly work within the fields of cancer cell biology and other disease models [55, 56], drug development [57], [58], [59] and cellular biology [60]. However, a notable exception is the work by Overmyer et al. in 2015. Rats selectively bred for high (HCR) vs. low running capacity (LCR) show greater metabolic health and longevity. To gain insights into putative mechanisms contributing to these phenotypic differences, Overmyer et al. performed proteomic/metabolomic analyses of muscle which showed increased fuel efficiency through utilisation of FA oxidation in HCR rats via a reduction in mitochondrial protein acetylation [61]. To add further insight, fluxomic analyses via infusion of U-13C, 15N-valine highlighted a component of differential BCAA degradation in response to exercise and that this may play a functional role in this mitochondrial protein acetylation [61]. This unique study highlights the potential added value of fluxomics within studies of exercise biomedicine in relation to metabolism, exercise performance and health.

The to-date lack of application within exercise biomedicine of fluxomics techniques may be reflected by several technical considerations. Firstly, in many applications fluxomics can be restricted by tracer choice, for example certain labelled tracers can only provide information on pathways through which they will be metabolised, the U-13C, 15N valine tracer used by Overmyer can only provide information on flux and activity on BCAA metabolism and other directly related metabolic networks, rather than large coverage of the flux across the whole metabolome. Therefore, the use of multiple substrate specific tracers may be needed to probe further. Some have attempted to overcome this by using more universal tracers such as 13-Carbon labelled glucose [62], but even this has its limits. One route through which fluxomics is likely to expand further in coming years is via the use of Deuterium Oxide (D_2_O; discussed more below), because water is involved in the vast majority of metabolic reactions and networks, providing this tracer has the potential to probe/label the vast majority of the metabolome and provide detailed fluxomic insight within a single experiment [63]. Secondly, the need for identification of positional labelling within fluxomics to determine routes of metabolic flux, adds another layer of complexity analytically to your standard metabolomics analyses. Many metabolomics analyses using LC-MS utilises soft ionisation techniques which only provide information on the molecular ion (M+H), to determine positional labelling of tracers further MS/MS based fragmentation is needed, extending analyses costs and time [49, 50]. Finally, one major issue to the limited implementation of wide coverage fluxomics is the vastly different rates through which some metabolite networks turnover, and the optimal sampling points needed to cover a wide range of targets. While clearly limited technically, fluxomics can provide key insights into a number of unknowns in the exercise biomedicine field and its application will continue to expand in this field [49, 50].

## Dynamic proteomics and exercise biomedicine – the future?

While dynamic proteomics is currently considered an exciting and niche technique, it, like fluxomics, is not new. Some of the first applications of these techniques were developed in the early 2000s using the Stable Isotope Labelling in Cell culture (SILAC) approaches, which helped to expand understanding of changes in protein dynamics in *in vitro* cell systems [64]. By replacing the cell medium with medium containing amino acids completely labelled with 15-Nitrogen, Deuterium, or 13-Carbon (stable isotope equivalents of organic elements Nitrogen, Hydrogen and Carbon) at single or multiple positions, it was possible to determine rates of incorporation of these labels within multiple new proteins using bottom-up proteomics [64, 65]. Pratt and colleagues developed this further through their pulsed SILAC approach, whereby cells labelled with stable isotope labelled media, were switched to unlabelled media for the duration of the experimental period, and the rate of labelled AA dilution in the cell proteins over time gave a dynamic view of protein turnover [66]. These approaches were rapidly developed for implementation in animal models, where it was possible to add 15N labelled algal cells to the diets of animals and take a similar analytical approach to measure incorporation and hence rates of whole proteome turnover [67]. This was a breakthrough; now not only could abundance/concentration of proteins with time be determined, but also how rapidly they were turning over to produce these changes.

These techniques were ideally suited to preclinical models, and thus, not easily translatable to the study of human metabolism *in vivo*, therefore an alternative method of labelling was devised using daily oral dosing with the stable isotope tracer deuterium oxide (D_2_O [68]). D_2_O rapidly equilibrates with body water (within ∼20 min in rodents [69]) and up to 1–2 h in adult humans [70] this creates a homogenous, slowly turning over, precursor pool available for use by multiple substrates. The deuterium from the body water can then be incorporated onto different substrates at stable C–H positions through biological reductions during *de novo* synthesis, including all the proteinogenic amino acids to different degrees [71, 72]. Therefore, using this approach of oral administration in humans, the amino acids would become labelled with deuterium from D_2_O, the labelled amino acids are then incorporated into proteins over time. A process that had previously been validated in animal and cell culture models using D_2_O [73], [74], [75], [76]. By taking serial samples over time Price was able to isolate peptides using LC-MS/MS technology and extract mass isotopomer abundance data relating to 114 unique proteins. By measuring the increase in enrichment of peptides by deuterium over time and comparing to the initial unlabelled isotopomer ratio for these peptides, rates of turnover were kinetically modelled providing one of the first *in vivo* measures of the dynamics of the human proteome [68]. Using this technique the turnover rate of proteins (such as albumin) was validated showing good agreement with rates measured using this new D_2_O approach to those previously reported using AA tracer techniques [68].

Since this seminal work there has been a rapid uptake of this unique method for human physiological research. Much of this ability to apply and accurately measure the dynamic proteome stems from the technological development of HR-MS equipment already described above, which whilst providing the rapid analyses time and increased depth of coverage of the proteome, also allows for the increased analytical resolution of multiple isotopologues (15N, 13C and deuterium), providing a more precise measurement of isotopomer abundance of each identified peptide and protein [48]. This ensures that experiments can be performed at comparably lower levels of D_2_O stable isotope tracer dosing (1–2 % in humans vs. 5–10 % in pre-clinical) in humans than were previously developed in pre-clinical models, thereby reducing costs and any adverse reactions to tracer administration [53]. In addition to these hardware developments, software has been critical to the development of dynamic proteomics approaches. To enable those without backgrounds in data science and mathematics to interpret and handle these data appropriately, a large focus has been placed on the development of open-source processing software with user friendly interfaces. Key examples of these developments can be seen through the work of Ping and Colleague with their software ProTurn [77], Price and colleagues with the ever-expanding software package DeuteRater [78] and Sadygov and Colleagues with D_2_Ome [79]. Sadygov in particular has thoroughly investigated key aspects of the optimal mathematical models for accurate and reproducible quantification of proteome turnover [80], methods for increasing proteome coverage through software processing pipelines [81] and optimal timepoint selection for experimental design and analyses [82], amongst many others [83, 84]. With these advances in processing, the ease in which dynamic proteomics can be incorporated into many routine human *in vivo* experiments has expanded rapidly, and these novel technologies are now finding greater application across multiple research fields as a result.

As is the case for fluxomics, whilst the benefits to the addition and potential of these dynamic proteomics approaches are evident, their application within the field of exercise remains in its early stages. The primary applications to date have been in the fields of health and disease [85] and drug development [86]. Of those that have been performed over the past decade utilising an exercise paradigm in their design have essentially involved some form of method development or proof-of-concept aspect to them, further supporting the power of the technique outside of key insights into detailed physiological change. Shankaran et al. [87]; applied these approaches within a small group of humans (n=3) comparing rested individuals who had undergone sprint interval training, highlighting that in response to sprint training rates of proteins involved with energy metabolism and cellular respiration were upregulated [87]. Additional work by Murphy et al. in 2018 showed that key energetic proteins, mitochondrial and myofibrillar assembly proteins are upregulated in response to resistance exercise in individuals under energy restriction [88]. These studies highlight the utility and applicability of the approaches in human muscle responses with increases in key pathways already known to be influenced through exercise, assisting to validate this approach within exercise studies. Yet one of the key aims of applying these techniques within these two studies was to assess the utility and validity of plasma protein turnover rates as a surrogate for muscle protein responses using these dynamic proteomic approaches [87, 88]. Further to these, work from the Burniston lab has also helped develop application of dynamic proteomics using D_2_O within humans, with a particular application to exercise, in an attempt to further validate its utility for translating key unknown aspects of exercise biomedicine. Specifically, their Absolute Dynamic Profiling Technique for Proteomics (Proteo-ADPT) allows the contributions of synthesis and degradation, to abundance measures in a single analysis [89, 90].

As discussed throughout this review, the field of OMICs has, through both *targeted* and *untargeted* approaches, enabled development of our existing knowledge of exercise biomedicine. However, to date, dynamic proteomics, as with fluxomics work, is limited in exercise biomedicine. In work that has been performed, findings generally are not that unexpected, following the trends that have been observed across many molecular or bulk protein turnover studies in response to exercise. Yet this may be owing to the generally low n (from n=2 to 8) associated with human application to date, and the poor overall coverage in terms of number of proteins quantifiable, hence the lack of novel outcomes observed. However, with development work currently being undertaken, these issues will likely be resolved soon. Finally, with novel techniques, there will always be a debate as to the best and most optimal methods as to the application of these dynamic OMICs. As static/multi/dynamic/‘OMIC data continue to be generated in exercise biomedicine, this will help drive development, application, and optimisation of these techniques further. Understanding the dynamics of metabolism is vital to ensure insights into exercise, health, therapeutics, and medicine continue to expand.
